# Early Isolates of SARS-CoV-2 Result in Different Pathogenesis in the Transduced Mouse Model of COVID-19

**DOI:** 10.3390/v14081769

**Published:** 2022-08-13

**Authors:** Sophie J. Smither, Sarah Kempster, Simon L. Priestnall, Alejandro Suárez-Bonnet, Helen Stapleton, Thomas R. Laws, Deborah Ferguson, Neil Almond, Mark S. Lever, E. Diane Williamson

**Affiliations:** 1 Chemical Biological and Radiological (CBR) Division, Defence Science and Technology Laboratory (Dstl), Salisbury SP4 0JQ, UK; 2 Division of Infectious Disease Diagnostics, National Institute for Biological Standards and Control (NIBSC), Medicines and Healthcare Products Regulatory Agency, Blanche Lane, South Mimms EN6 3QG, UK; 3 Department Pathobiology & Population Sciences, The Royal Veterinary College, Hawkshead Lane, North Mymms, Hatfield AL9 7TA, UK

**Keywords:** SARS-CoV-2, COVID-19, isolates, mouse model, transduction, virulence

## Abstract

A transduced mouse model of SARS-CoV-2 infection was established using Balb/c mice. This was achieved through the adenovirus-vectored delivery of the hACE2 gene, to render the mice transiently susceptible to the virus. The model was characterised in terms of the dissemination of hACE2 receptor expression, the dissemination of three SARS-CoV-2 virus variants in vivo up to 10 days following challenge, the resulting histopathology and the clinical signs induced in the mice. In transduced mice, the infection was short-term, with a rapid loss in body weight starting at day 2 with maximum weight loss at day 4, followed by subsequent recovery until day 10. The induced expression of the hACE2 receptor was evident in the lungs, but, upon challenge, the SARS-CoV-2 virus disseminated beyond the lungs to spleen, liver and kidney, peaking at day 2 post infection. However, by day 10 post infection, the virus was undetectable. The lung histopathology was characterised by bronchial and alveolar inflammation, which was still present at day 10 post infection. Transduced mice had differential responses to viral variants ranking CVR-Glasgow 1 > Victoria-1 > England-2 isolates in terms of body weight loss. The transduced mouse model provides a consistent and manipulatable model of SARS-CoV-2 infection to screen viral variants for their relative virulence and possible interventions.

## 1. Introduction

Since late 2019/early 2020, the pandemic caused by the Severe Acute Respiratory Syndrome–Coronavirus 2 (SARS-CoV-2) has affected every continent and nearly every country. Worldwide, the COVID-19 pandemic caused over 500 million cases and more than 6 million deaths by April 2022 [1,2] and these figures may well be under-estimates. Unlike the SARS outbreak in 2003, which occurred for a relatively short 9 months and affected 8422 people in 32 countries, of whom 919 died, and was self-limiting [3], SARS-CoV-2 is still very active in terms of both transmission and mutation, with variants arising globally and continuing to circulate and infect beyond the initial region or country [1]. Vaccination against SARS-CoV-2 has been extremely successful in preventing hospitalisation or death, with some estimates indicating that over 10 million lives were saved in the first year of vaccination [4], across the world, but the remarkable ability of SARS-CoV-2 for human-to-human transmission and its capacity to mutate to maintain transmission rates [5,6] remain a global concern.

SARS-CoV-2 is an enveloped RNA virus whose mechanism of action consists of the interaction of the viral spike (S) protein with the angiotensin 1 converting enzyme 2 (ACE2) receptor of the host [7], which is displayed on the cell surface of major human tissues and organs [8]. The remarkable ability of SARS-CoV-2 to infect is attributed to the high binding affinity of the S protein for ACE2, with the subsequent cleavage of the S protein by cellular proteases such as TMPRSS2, leading to the fusion of cellular and viral membranes and cell entry [9]. Since its first identification in late 2019/early 2020, SARS-CoV-2 has developed a number of point mutations and deletions in the S protein, some of which are within its receptor-binding domain (residues 319–541) [9,10,11] and in upstream (NTD 13–318) and downstream regions, such as 613–705 of Subdomain 1 (S1), part of the S1/Subdomain 2 (S2) junction and a small section on the S2 side. These mutations have had the effect of increasing the affinity of the RBD for hACE2 even further, such that more infectious variants have arisen [12] following passage through regional, national and international populations.

SARS-CoV-2 mutations led to the development, initially, of four classes of viral variants, designated Alpha, Beta, Gamma and Delta, that arose in the UK, South Africa, Brazil and India, respectively, in the period December 2020–May 2021 and were classified as variants of concern (VOC) by the WHO [1]. Subsequently, the Lambda and Mu variants were detected in Peru and Colombia in June and August 2021, respectively, and have been monitored by the WHO as variants of interest [1]. Descendants from these lineages continue to be monitored in countries around the world by the WHO. The Omicron variant (BA.1 lineage), with 32 mutations in the S protein, subsequently emerged in southern African countries in November 2021, with significantly increased transmissibility and a higher risk of vaccine escape [13,14]. More recently, further sublineages of the Omicron variant have emerged (BA.2–BA.5), with amino acid differences appearing in the spike protein and other proteins. These variants are being closely monitored by the WHO [15] since they appear to have a growth advantage and are inherently more transmissible compared to the BA.1 VOC.

Here, we have gone back to three of the original clinical isolates of SARS-CoV-2 collected early in the pandemic, namely Victoria-1 (Australia/VIC1/2020), England 2 (England/02/2020) and Glasgow 1 (Glasgow/01/2020), to establish a murine model of the original virus. The first isolate, Victoria-1, has no known coding mutations relative to Wuhan-Hu-1 (NC_045512), while the England 2 stock used has a furin-cleavage site mutation within the S glycoprotein [J. Findlay unpublished observation]; this a common feature in laboratory-propagated stocks [16], and Glasgow 1 has a D614G mutation in S2 of the S protein and P323L in nsp12 [17].

The need for animal models of COVID-19 to screen the parent virus and variants and to assess potential therapies is apparent. Only some animal species are naturally susceptible to SARS-CoV-2. This is likely due to the possession of an ACE2 receptor with sufficient homology to the human receptor, to allow the binding of the S protein of the virus [18]. Naturally susceptible species include hamsters, some non-human primates, deer, mink and raccoons [19]. Although mice are not naturally susceptible, this species can either be genetically modified to permanently express the hACE2 receptor [20] or it can be transduced through the adenoviral vector delivery of the hACE2 receptor gene, to cause a short-term expression of hACE2 R [21], which renders mice transiently susceptible and provides a model of infection ideally suited for the rapid screening of isolates and potential prophylaxes/therapies.

In this study, we describe the establishment of a transduced murine model for COVID-19 in Balb/c mice and use this model to compare the relative pathogenesis of three original clinical isolates of SARS-CoV-2. The transduced murine model described provides a rapid but consistent model which has the potential to discriminate between different viral variants and also to screen potential prophylaxes and therapies for COVID-19.

## 2. Materials and Methods

### 2.1. Adenovirus Vector Production and Confirmation of Receptor Expression

The adenovirus vector was constructed through the insertion of the transgene encoding the 805-amino acid sequence [10] of the hACE2 receptor protein plus stop codon into the E1-deleted region of replication-defective E1/E3-deleted adenovirus type 5 produced in HEK293 cells using plasmid DNA construct REF: R800 (Oxgene, Oxford, UK) Protein Bank accession no. for hACE2 is 1R42. The resulting construct was of 33,363 bp. An ‘empty construct’ was prepared in exactly the same replication-defective E1/E3-deleted adenovirus type 5 adenovirus, but without the transgene insert. Expression of the hACE2 is under the control of a cytomegalovirus (CMV) promoter. The Ad5-CMV-hACE2 vector was subjected to two rounds of clonal selection and amplification with caesium chloride purification. The Ad5h-CMV-hACE2 vector was tested in vitro for expression by transfection of A549 cells (Human Caucasian lung carcinoma cells (ECACC reference number 86012804)); after 48 h, expression was confirmed by Western blot. The Ad5-CMV-hACE2 construct was supplied at a concentration of 1.61 × 10^10^ 50% tissue culture infectious dose (TCID_50_)/mL or 1.24 × 10^12^ viral particles (VP)/mL by PicoGreen assay, whilst the empty construct was supplied at 2.38 × 10^11^ TCID_50_/mL. Both constructs were diluted in PBS to deliver 2.5 × 10^8^ TCID_50_ in 50 µL/mouse intranasally.

A549 cells were cultured under standard conditions. A549 cell line was tested for optimal multiplicity of infection (MOI) for transduction using a control (GFP) Ad5-Ad5-hACE2 vector was used to transduce cells at MOI 50, 100 and 500. After 48 h, cells were harvested and the cell pellet was prepared for Western blot analysis. Western blot analysis was performed using two antibodies for human ACE2 detection (Abcam #108252 and Abcam #15348) and tubulin (as loading control); a recombinant human ACE2 protein was used as positive control (Abcam #151852). Membranes were cut by the 75 kDa band to allow the detection of both hACE2 and tubulin in the same sample. Detection was carried out via tetramethylbenzidine colorimetric output.

### 2.2. Cells and Viruses

Vero C1008 cells (ECCAC, Salisbury, United Kingdom, catalogue no. 85020206) were maintained in L15 tissue culture media (TCM; Sigma, Gillingham, UK) supplemented with 2% fetal calf serum, 1% L-glutamine, and 1% penicillin/streptomycin.

SARS-CoV-2 England-2 (hereinafter Engl2), isolated from a 23-year-old male in January 2020 (GISAID Accession ID EPI_ISL_407073), was kindly provided by the UK Health and Security Agency, Porton Down. Passage 2 stocks were grown in Vero C1008 cells infected at MOI = 0·01, harvested after 3 days and clarified by centrifugation (350× *g*, 5 min in a Thermo-Scientific Sorvall Legend ×1 centrifuge).

SARS-CoV-2 Victoria-1 (BetaCoV/Australia/VIC1/2020, Genbank: MT007544) (hereinafter Vic1) was provided by the UK National Institute for Biological Standards and Control (NIBSC) Research Reagents Repository, with thanks to D. Mike Catton, Melbourne, Australia. The original virus (passage 3) was received from Dr Mike Catton, Victorian Infectious Diseases Reference Laboratory, Melbourne, from the first patient diagnosed with COVID-19 in Australia [22]. The virus was supplied by the Centre For AIDS Reagents (CFAR), NIBSC, as passage 4 grown in the VeroE6/TMPRSS2 cell line (CFAR #100978).

SARS-CoV-2 CVR-Glasgow1 (a D614G mutant, CFAR# 1000998, Genbank: MT882022) (hereinafter Gla1) was also provided by the UK NIBSC Research Reagents Repository, with thanks to Prof Arvind Patel, The MRC-University of Glasgow Centre for Virus Research [23]. The virus was supplied by CFAR, NIBSC as passage 3. One high-sfrequency SNP was not present in the consensus sequence provided by the depositor (passage 2), at position 26258, resulting in Val→Ala substitution in the E gene.

All manipulation of SARS-CoV-2 was performed in an ACDP Containment Level 3 laboratory.

### 2.3. Animals

Animal studies were performed in accordance with the United Kingdom Scientific Procedures Act (Animals) 1986 and the United Kingdom Codes of Practice for the Housing and Care of Animals Used in Scientific Procedures, 1989. Female Balb/c mice (Charles River, UK) aged 6–8 weeks were housed in groups of 5 or 6 and had ad libitum access to food and water in an ACDP Containment Level 3 half-suited rigid isolator. After challenge, mice were weighed daily and observed twice daily for clinical signs.

### 2.4. Study Designs

In both studies, mice were transduced with 2 × 10^8^ tissue culture infectious dose (TCID_50_) Ad5-CMV-hACE2 by the intranasal (i.n.) route, 25 µL per nostril, under light anaesthesia with isoflurane. Control mice in both studies were administered empty vector. Five days after transduction, mice were challenged with 50 µL SARS-CoV-2 suspension i.n. (25 µL per nostril), under light anaesthesia. After challenge, mice were weighed daily and checked at least twice daily and all clinical signs were recorded. (Weight loss of 30% or more would qualify as a humane end-point and clinical signs or combinations of clinical signs such as loss or reduction in mobility, rapid breathing, closed eye(s), posture and coat condition.) At pre-determined time-points, mice were humanely killed and cardiac puncture was performed under terminal anaesthesia (isoflurane followed by cervical dislocation). Post-mortems were performed with the removal of lungs, heart, brain, spleen and kidney.

In Study 1, individual groups of 6 mice were challenged at one of 3 dose-levels in the range 10^2^–10^4^ TCID_50_ with Vic1 or 10^3^–10^5^ TCID_50_ Engl2 and all mice were humanely killed on day 4 post-challenge (Table 1).

In Study 2, groups of 5 mice were challenged with a single dose of 10^5^ TCID_50_ Vic1 and humanely killed on days 2, 3, 4, 7 and 10 post challenge. In addition, groups of 5 mice were challenged with escalating dose-levels of Gla1 in the range 10^2^–10^4^ TCID_50_ and all of these mice were humanely killed on day 4 post challenge (Table 1).

### 2.5. Viral Enumeration Assay

Enumeration was carried out via a TCID_50_ assay: virus was serially diluted ten-fold in 96-well plates of Vero C1008 cells and after 3–6 days at 37 °C, wells at each dilution were scored for the presence of cytopathic effects by microscopic observation. Titre was determined by Reed and Muench calculation [24]. For enumeration of lung titres, segments of lung were homogenised in 1 mL tissue culture medium (TCM) and a 1:10 dilution was made prior to TCID_50_ assays being performed.

### 2.6. Immunoassay

Animals were blood-sampled by cardiac puncture under terminal anaesthesia prior to humane euthanasia by cervical dislocation and the sera were collected for virology and immunoassay. The competitive ELISA (GenScript Biotech UK Ltd., Oxford, UK) was used to determine whether animals challenged with SARS-CoV-2 had developed antibody against the receptor binding domain (RBD) of SARS-CoV-2. The kit was used according to the manufacturers’ instructions. This assay is a competitive ELISA that detects total antibody and results are presented as a percentage inhibition of binding of RBD to hACE2.

### 2.7. Histopathological Analysis

The lungs were removed from mice post-mortem, formalin fixed and embedded in paraffin wax before sections of 4 µm were taken and mounted on poly-L-lysine-coated slides (VWR, Leicestershire, UK). Prior to manual staining, sections were de-waxed with xylene (Fisher Scientific, Loughborough, UK) and re-hydrated via graded ethanol: water solutions (Fisher Scientific, Loughborough, UK). Lung sections were stained in accordance with standard histological procedures. H&E sections were evaluated for a range of histopathological changes and assigned a score (0–4 for absent, minimal, mild, moderate and marked, respectively) for each variable, independently assessed by two board-certified veterinary pathologists blinded to the experimental details. The following histopathological variables were assessed semi-quantitatively from each lung section: intra-alveolar haemorrhage, intra-alveolar oedema, intra-alveolar fibrin, alveolar inflammation, alveolar wall necrosis, presence of hyaline membranes, bronchiolar inflammation, type II pneumocyte hyperplasia, vascular necrosis, fibrosis and haemosiderin. In addition, the percentage of tissue affected per lung was recorded. Two sections of lung from different lobes from all mice, in both studies, were subjected to detailed analysis.

### 2.8. Immunohistochemical Analyses

Immunohistochemical staining was performed using the Leica Bond RXm automated stainer, Bond Polymer Refine staining system (Leica Microsystems DS9800) and associated Leica Bond consumables. Onboard de-waxing was performed in accordance with the standard Leica Bond protocol and staining was undertaken using IHC Protocol B with the following adaptations: additional non-specific block prior to primary antibody incubation (20% normal goat serum (Biorad, Watford, UK), 1× Casein (Vector Labs, Burlingame, CA, USA) in PBS) and extended haematoxylin staining time (10 min). Antigen unmasking to allow antibody binding was undertaken using 40 min heat retrieval in solution 1 (Leica BondMax, Milton Keynes, UK). Antibodies were diluted to their optimal staining concentration in blocking buffer: SARS-CoV-2 Nucleoprotein antibody, rabbit polyclonal (Sino Biological, Eschborn, Germany) 1:200 or ACE2 rabbit polyclonal (Sigma, Gillingham, UK), 1:200.

### 2.9. RNA Extraction and PCR

Organs (lung, brain, spleen and kidney) were dissected and collected into RNAlater (ThermoFisher, Paisley, UK) and stored at −80 °C until use. Thawed organs were homogenised in 1 mL tissue culture medium (TCM) and then 140 µL used for RNA extraction. RNA extraction was performed using the QIAamp Viral RNA kit (Qiagen, Manchester, UK). Alongside organ samples, virus was serially diluted in TCM or naïve lung homogenate, and RNA extractions were performed in duplicate to create a standard curve in the range 5 × 10^5^ down to 0.5 TCID_50_, to calibrate the qRT-PCR as TCID_50_.

The SARS E-gene PCR was performed on a QuantStudio (TM) 7 Flex System. The E gene PCR assay is a previously published assay [25] targeting a section of the SARS-CoV-2 E gene. Next, 96-well PCR plates containing the mastermix (20 µL/well) for the E gene assay were prepared in advance and frozen (−20 °C). The mastermix consisted of 4 × FastVirus Mastermix (ThermoFisher), 400 nM E gene forward primer (5′-ACAGGTACGTTAATAGTTAATAGCGT-3′), 400 nM of E gene reverse primer (5′-ATATTGCAGCAGTACGCACACA-3′) and 200 nM of E gene probe (5′ P1 FAM-ACACTAGCCATCCTTACTGCGCTTCG-BHQ-3′). Additionally, included in the mastermix was an MS2 internal control assay comprising 80 nM MS2 forward primer, 80 nM MS2 reverse primer and 160 nM MS2 probe. When ready for use, the plate was thawed and centrifuged. In total, 5 µL of the test sample was added to each well as required. The PCR plate was run on a QuantSudio 7 Flex machine using the following amplification programme: 55 °C for 10 min; 94 °C for 3 min; for 45 cycles; 94 °C for 15 s; 58 °C for 30 s. Cycle threshold (Ct) values were recorded for each sample. The Ct value is designated at the end of the amplification cycle where the end point fluorescence crosses the threshold (designated by the QuantStudio Flex software, V1.7).

### 2.10. Statistical Analysis

Statistical analyses were performed using SPSS (IBM, V27.0) and charts were generated using Graphpad PRISM (V7.0). The validity of univariate and repeated-measures linear models was assessed using diagnostic residual plots. Viral load data were transformed by the logarithm of 10 to better fit the test requirements. Where pairwise models were performed, the *p*-values were adjusted using Bonferroni’s correction.

## 3. Results

### 3.1. In Vitro Expression of hACE2 in Transfected A459 Cells

**Figure 1:**
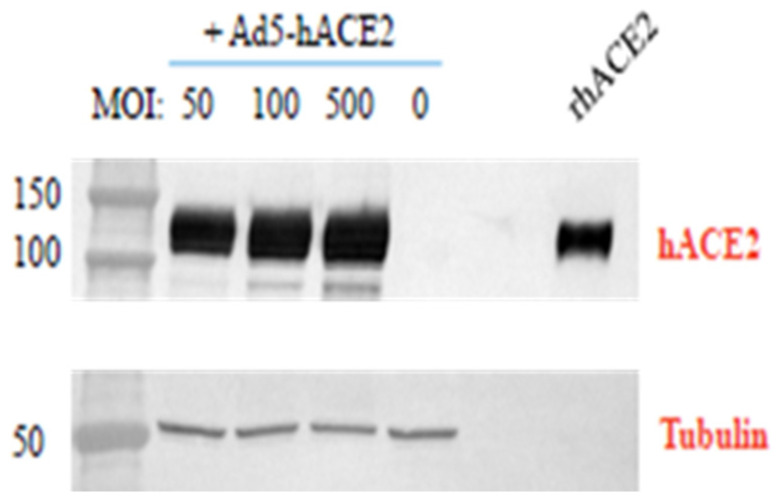
Western blot analysis of hACE2 expression in transfected A549 cells at 48 h. After 48 h of culture, A549 cells were harvested and the cell pellet was prepared for Western blot analysis, utilising a monoclonal antibody for human ACE2 detection and an anti-tubulin antibody (as a gel-loading control); a recombinant human ACE2 protein (125 ng) was used as a positive control. Non-transduced cell lysates were negative for hACE2 expression and tubulin was detected in all cell lysates at the expected size of approximately 50 kDa (the band is composed of alpha-tubulin (52 kDa) and/or the heterodimer (55 k Da)).

A549 cells were used to test the expression of hACE2 in vitro, after transduction with the Ad5-CMV-hACE2 vector. Initially, a control (GFP) Ad5 was used to establish an optimal multiplicity of infection (MOI) for transduction. Subsequently, the Ad5-CMV-hACE2 vector was used in A549 cells at MOIs of 50, 100 and 500. Figure 1 shows an increasing signal for hACE2 expression with increasing MOI in the transfected A549 cells.

### 3.2. Weight Change, Lung Viral Load and Immunohistochemical Analysis of Lung Tissue in Transduced Mice Following Infection with Different Variants of SARS-CoV-2

**Figure 2:**
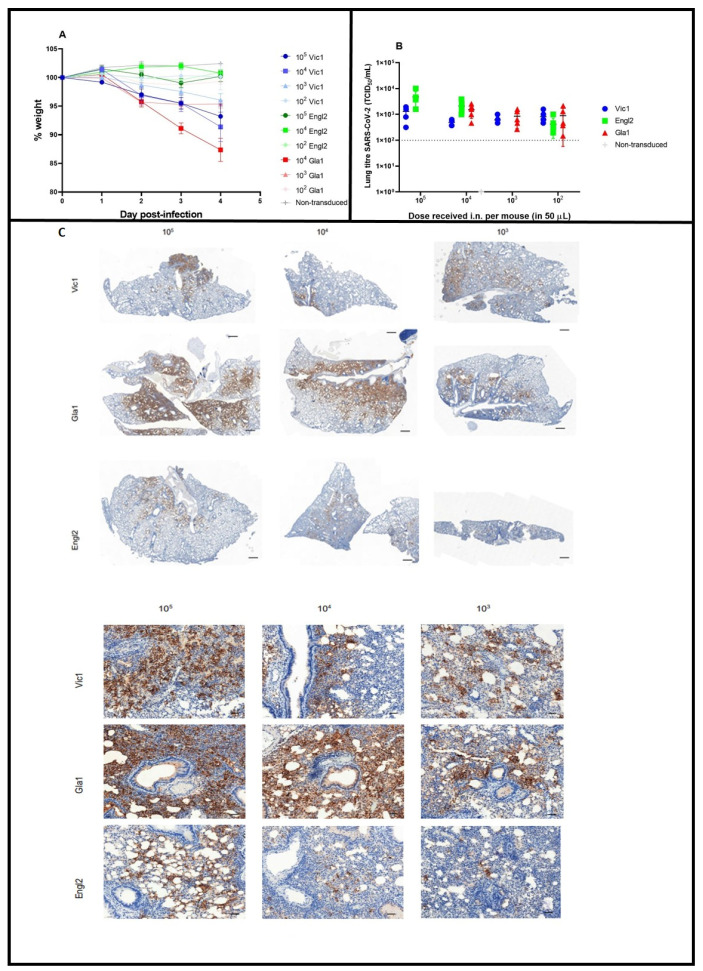
Variation in weight change, lung titres and presence of viral nucleoprotein at day 4 post-infection after challenge with different doses and strains of SARS-CoV-2. Groups of five or six mice were transduced with Ad5-CMV-hACE2 and five days later infected with the indicated dose-level (TCID_50_ in 50 µL i.n.) of SARS-CoV-2 Vic1 (blue symbols and lines), Engl2 (green symbols and lines) or Gla1 (red symbols and lines). Mice transduced with empty vector and challenged with a high dose of SARS-CoV-2 Vic1 were negative controls (grey lines and symbols). (**A**) Mean group weight (+/− SEM as error bars) post infection as a percentage where day 0 weight is 100%. (**B**) Titre of viable SARS-CoV-2 in lungs at day 4 p.i. Individual values for each mouse shown with a line at the mean. Limit of detection = 100 TCID_50_ (dotted line). (**C**) Viral nucleoprotein was detected by IHC in lung sections at day 4 p.i. from mice infected with dilutions of Vic1, Gla1 or Engl2. Scale bars represent 500 µm for low-magnification and 100 µm for high-magnification images.

Groups of Ad5-CMV-hACE2-transduced mice were infected five days later with different doses of different variants of SARS-CoV-2 (Table 1). Doses were based on the highest dose achievable given the stock concentration and the volume that could be administered to mice, and then ten-fold dilutions were conducted. No clinical signs were observed; however, mice infected with 50 µL containing 2.1 × 10^4^ TCID_50_ SARS-CoV-2 Gla1 showed the greatest weight loss, with a mean weight loss of around 5% a day between days 2 and 4 (Figure 2A). Mice challenged with the middle dose of SARS-CoV-2 Gla1 (2.8 × 10^3^) and those challenged with the two higher doses of SARS-CoV-2 Vic1 (1.6 × 10^5^ and 4 × 10^4^) also showed some weight loss (between 5 and 10%), (Figure 2A). However, minimal weight loss was observed in mice challenged with doses of 10^2^ SARS-CoV-2 Vic1 or SARS-CoV-2 Gla1 and all doses of SARS-CoV-2 Engl2; their weight was similar to that of controls, mice transduced with an empty vector and then challenged with a high dose of SARS-CoV-2 Vic1 (Figure 2A).

In terms of lung counts at day 4 p.i., all mice that were transduced with the Ad5-CMV-hACE2 vector and challenged with any of the three strains of SARS-CoV-2 had viable virus in their lungs (Figure 2B). No viable virus was recovered from the lungs of mice transduced with the empty vector, or non-transduced and subsequently challenged with the highest dose of SARS-CoV-2 (Figure 2B). Transduced mice challenged with the Engl2 strain showed a dose–response effect, with higher lung titres resulting from higher exposure. Mice challenged with Vic1 or Gla1 had consistent viral titres in lungs, irrespective of the dose delivered (Figure 2B).

Immunohistochemistry (IHC) analysis showed evidence of viral nucleoprotein in lung tissues at day 4 p.i. irrespective of the dose or virus isolate. Nucleoprotein staining decreased when the virus dose decreased (Figure 2C). The nucleoprotein appeared to be associated with areas surrounding the larger airways within the centre of the lung lobe and disseminating towards the periphery of the lobe with reduced staining at the edges of the lobe. The staining appeared as more discrete patches as the viral dose decreased, which is associated with pneumocytes.

### 3.3. Weight Change, Lung Viral Load and Immunohistochemical Analysis of Lung Tissue in Transduced Mice at Different Times after Infection

**Figure 3:**
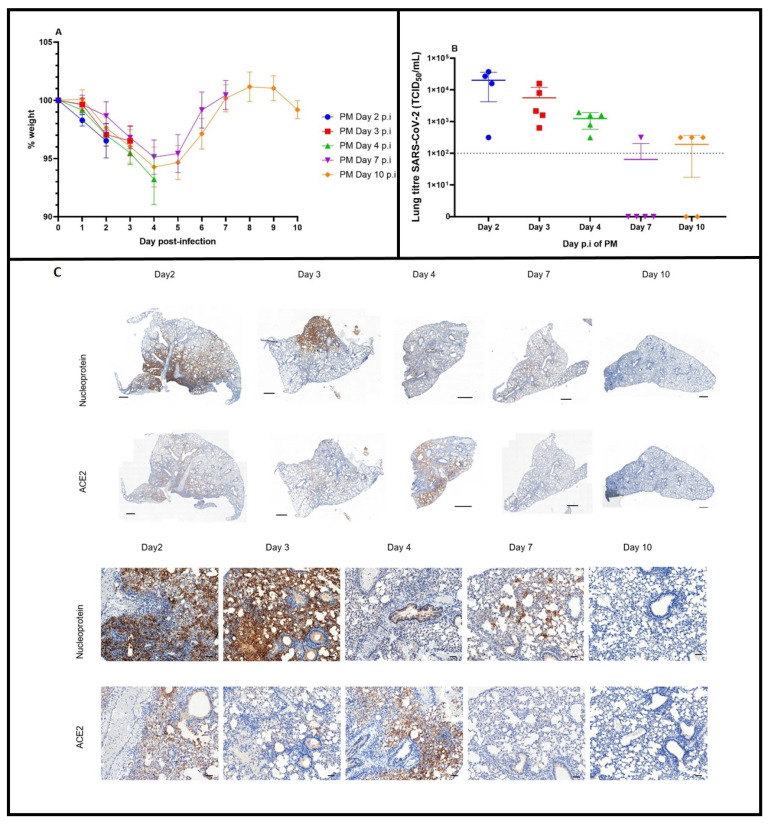
SARS-CoV-2 Vic1 infection in mice over time. Groups of five mice were transduced with Ad5-CMV-hACE2 and five days later infected with 1.6 × 10^5^ TCID_50_/50 µL of SARS-CoV-2 Vic1 by the i.n. route. At day 2 p.i. (blue symbols and lines), day 3 (red symbols and lines) day 4 (green symbols and lines), day 7 (purple symbols and lines) or day 10 (orange symbols and lines), a group of mice were humanely killed. (**A**) Mean group weight (+/− SEM as error bars) post-infection as a percentage, where day 0 weight is 100%. (**B**) Titre of viable SARS-CoV-2 in lungs at different time-points p.i. Individual values for each mouse shown with a line at the mean. Limit of detection = 100 TCID_50_ (dotted line). (**C**) IHC staining for SARS-CoV-2 nucleoprotein or ACE2 protein in mouse lung at 2, 3, 4, 7 and 10 days p.i. with 10^5^ TCID_50_ Vic1. Nucleoprotein staining was detected on days 2 and 3 p.i. and reduced on days 4, 7 and 10 p.i. ACE2 was detectable in lung tissue on days 2, 3, and 4 and not on days 7 and 10. Scale bars represent 500 µm in macroimages and 100 µm in magnified images.

To characterise the time-course of SARS-CoV-2 infection in Ad5-CMV-hACE2-transduced mice, groups of five animals were challenged with a dose of 1 × 10^5^ TCID_50_ SARS-CoV-2 Vic1 by the i.n. route, weighed daily and humanely killed at 2, 3, 4, 7 and 10 days p.i. All mice were losing weight by day 2, and this reached a peak at day 4 p.i. After day 4 p.i., mice started to recover body weight and had returned to pre-challenge levels by day 7 p.i. (Figure 3A).

The amount of viable virus recovered from the lungs was the highest at day 2 p.i. and then decreased on days 3 and 4 p.i. By day 7, and also at day 10 p.i., in some cases no viable virus was recovered from lungs, and others had low levels (Figure 3B).

Immunohistochemical staining revealed that nucleoprotein was present at days 2, 3 and 4 p.i. and reduced at days 7 and 10 p.i. ACE2 staining was present at detectable levels on days 2, 3 and 4 p.i. and absent on days 7 and 10 p.i. (Figure 3C).

### 3.4. Statistical Analysis of Weight Change and Viral Titres Post Infection

A repeated-measures linear model was constructed to better understand the roles of the viral strain and dose level (log10-transformed as a continuous variable) on weight change. This model indicated a high probability for a complex interaction of the dose level and strain on weight change post infection (*p* = 0.004). Separate pairwise models were used to compare strains. The statistical models indicated that all strains were likely to have different dose–effect profiles. These comparisons included: Vic1 compared to Engl2 (*p* = 0.561), Vic1 compared to Gla1 (*p* = 0.231) and Engl2 compared to Gla1 (*p* < 0.003).

A linear model was constructed to characterise the data for viral titres in lungs at day 4 p.i. The model indicated that the effect of the exposure dose level on viral titre was different for the virus strains (*p* < 0.001). This model was partitioned to consider the differences between each strain. When only Vic1 and Engl2 were included in the model, the differing effect of the dose level between strains was evident (*p* < 0.003). When only Vic1 and Gla1 were considered, no differences were observed between strains (*p* = 0.519). When only Engl2 and Gla1 were included in the model, the differing effect of exposure dose level between strains was also evident (*p* < 0.003), and there was no evidence for differences between strains (*p* = 0.297). Collectively, this analysis suggests that increasing exposure dose level influences viral burden at 4 days p.i. and this effect is more pronounced in the mice infected with the Engl2 strain.

### 3.5. Quantitative PCR Indicates Widespread Viral Dissemination

RNA was extracted from the organs removed at day 4 p.i. from transduced mice challenged with either Vic1 (4 × 10^4^ TCID_50_/50 µL i.n.) or Gla1 (2.1 × 10^4^ TCID_50_/50 µL) at day 4 p.i., and analysed by qRT-PCR analysis. Relative Ct values were read from standard curves for conversion to TCID_50_ values.

Viral RNA was detected in all lung, spleen, kidney brain and heart samples (data not shown). Mouse lungs had the highest amount of viral RNA with the least variation, as indicated by the low standard deviation and mean Ct values (mean = 13.366 and SD = 0.322 for all). Spleens, kidneys, brains and hearts all had less RNA, as shown by the higher mean Ct values in the range of 26–30 for all organs and both isolates. Ct values indicated higher amounts of RNA in spleens and hearts from mice infected with SARS-CoV-2 Gla1 compared to those infected with SARS-CoV-2 Vic1. For brains, the converse held (more RNA in Vic1-infected mice), and for kidneys, the levels were very similar.

### 3.6. Antibody Response to RBD

**Figure 4:**
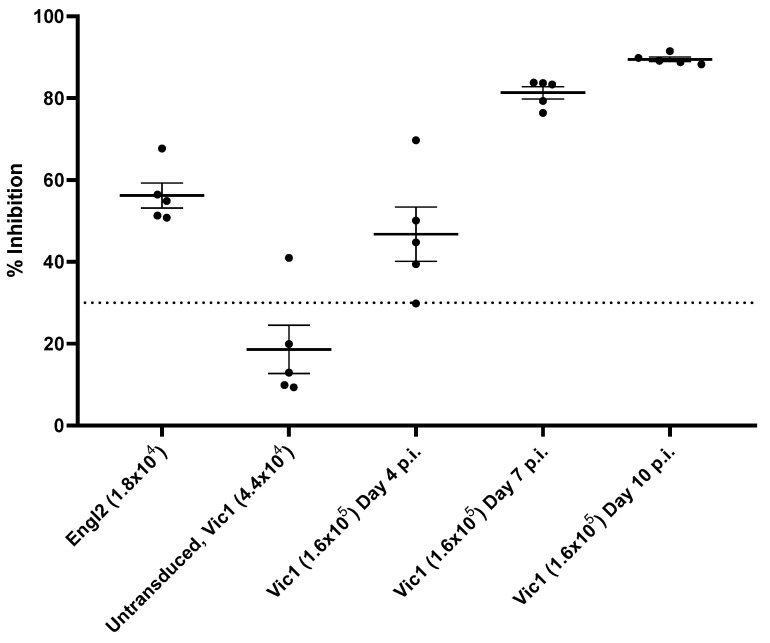
Infected mice developed an antibody response to RBD. Mouse serum samples taken at termination on days 4, 7 or 10 p.i. were tested for their ability to compete with wild-type RBD for binding to the ACE2 receptor. Dotted line represents the cut-off of the assay to define whether it is positive (>30%) or negative (<30%) according to the manufacturers’ instructions.

Serum samples taken from mice at days 4–10 p.i. with SARS-CoV-2 Vic1 were tested by competitive ELISA to bind to the receptor-binding domain (RBD) of SARS-CoV-2 (Figure 4). Whilst serum from non-transduced mice, which was challenged with Vic1, showed no competitive binding, serum samples from transduced mice challenged with either Engl2 or Vic1 and humanely killed at day 4 p.i. inhibited 50–60% binding, whilst samples from Vic1-challenged mice that had been humanely killed at day 7 or 10 p.i. achieved 80–90% inhibition (Figure 4).

### 3.7. Histopathological Analysis

**Figure 5:**
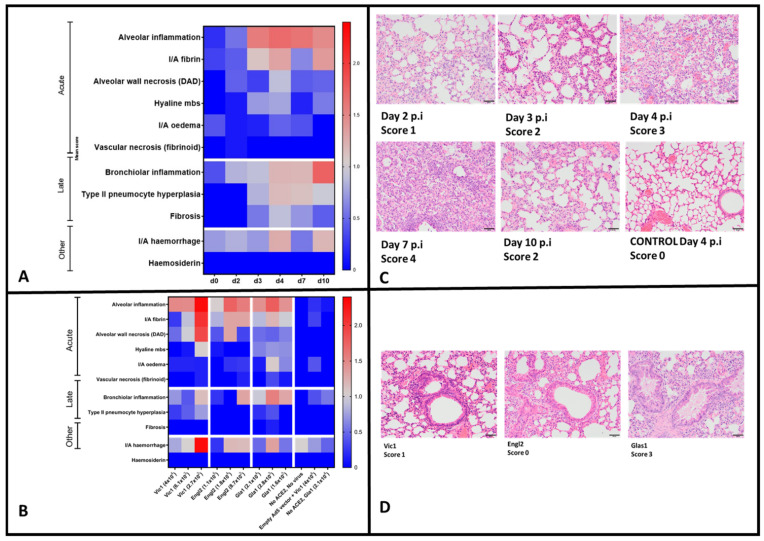
Histopathology in lungs from mice infected with SARS-CoV-2. For all mice, lungs were scored based on the histopathological criteria indicated. The mean group score multiplied by the percentage of tissue affected was determined and is shown as a heat map ranging from low (blue) to higher values (red). (**A**) Groups of five mice were transduced with Ad5hACE2 and five days later infected with a single dose of SARS-CoV-2 Vic1. Mice were humanely killed on different days (0, 2, 3, 4, 7 and 10), as indicated on the *x*-axis and lungs collected in formalin for histological analysis. (**B**) Groups of five or six transduced mice were infected with the indicated dose of SARS-CoV-2 Vic1, Engl2 or Gla1. Control mice were also treated as indicated. All mice were humanely killed on day 4 post infection and lungs were collected in formalin for histological analysis. (**C**) Groups of five transduced mice, challenged with 10^5^ TCID_50_ SARS-CoV-2 Vic1, were humanely killed at days 2–10 post infection (p.i.), with lung histopathology examples of haematoxylin and eosin staining. (**D**) A comparison of the bronchiolar inflammation which had developed by day 4 p.i. in mice infected with 10^4^ TCID_50_ SARS-CoV-2 Vic1, Engl2 or Gla1 was carried out, showing that Gla1 induced more severe inflammation than Vic1 or Engl2. For c and d Images at ×200; bar = 50 µm.

To characterise lung pathology after infection with the highest dose of SARS-CoV-2 Vic1 (10^5^ TCID_50_) over time, sequential post-mortems at days p.i. 2, 3, 4, 7 and 10 were performed. Multiple histopathological changes in the lung were assessed and the average score for each individual mouse was multiplied by the percentage of tissue affected and the group mean for each criteria (categorised as acute or late phase inflammatory response or other) was evaluated (Figure 5A). Over time, an increase was seen in alveolar inflammation from day 2 p.i., increasing daily through to day 7 p.i., and decreasing by day 10 p.i (Figure 5A,B).

All other indicators of acute inflammatory response remained low throughout, with the exception of fibrin, which was at higher levels on days 4 and 10 p.i. (Figure 5A). Haemorrhage was also more abundant at these time-points (Figure 5A). In terms of late response, bronchiolar inflammation increased over time, leading to the highest score on day 10 p.i. and Type II pneumocyte hyperplasia also appeared at later time-points (days 4 and 7) (Figure 5A).

When looking at the histological features of the lungs of mice infected with different doses and variants of SARS-CoV-2, there was no clear dose-related pattern (Figure 5C). After infection with SARS-CoV-2 Vic1, lung features, particularly those associated with an acute response and haemorrhage, were paradoxically the highest in the mice receiving the lowest dose of the virus (Figure 5C). However, bronchiolar inflammation was high at each dose in mice infected with SARS-CoV-2 Gla1 compared with those infected with SARS-CoV-2 Engl2 and SARS-CoV-2 Vic1 (Figure 5C,D).

## 4. Discussion

In response to the pandemic, many animal models have been established to evaluate prophylaxes and therapies which can provide clinical benefits for SARS-CoV-2 infection [26]. The model of infection should authentically replicate features of human infection in order to have utility.

Here, we followed the approach originally used by Hassan et al. [20] to establish a Balb/c mouse model of COVID-19, by sensitising mice to SARS-CoV-2 with an adenoviral vector expressing hACE2. A previous work employed a novel E1-deleted adenovirus backbone, which also lacked the inverted terminal repeat sequence and packaging signals, and which was co-transfected with a shuttle vector carrying the hACE2 gene into HEK 293 cells [27], whilst our methodology comprised a standard viral genome/shuttle plasmid recombination approach, but also with the transfection of HEK 293 cells. Here, we have also shown that the transduction of naïve mice with an adenovirus vector carrying the hACE2 gene renders mice consistently susceptible to SARS-CoV-2 infection. One of the potential disadvantages of this model is that the induced susceptibility is too transient to be useful. However, here, we have shown that SARS-CoV-2 infection in the model lasts for 7 days, after which the virus is not detectable in organs. Moreover, the 12 days required (5 days for transduction and 7 days for infection) in the model is entirely appropriate to screen for vaccine/therapeutic efficacy, since the effect of the infection is so clear-cut in the transduced mouse. The model was evaluated to 10 days post infection to assess any longer-term pathologies associated with disease and thereby provide a model to assess any impact of interventions.

A major advantage of the transduction approach is that starting with a naïve mouse, an animal model can be built rapidly by transducing susceptibility to mimic the human condition through the simple administration of a viral vector, which delivers a gene encoding the appropriate receptor. Whilst we and others [21] have shown here that this works well for SARS-CoV-2, in theory this approach would work for any virus for which the human receptor is known. Furthermore, the ability to use transduced immune-deficient mice such as Rag1 knockout mice on the same Balb/c background enables the model to be adapted to interrogate components of the immune response, adoptive transfer can also be utilised [28]. Herein, we demonstrate seroconversion after infection through the detection of antibodies against the RBD of SARS-CoV-2 over time, which supports the use of this model in serological studies.

The transduced mouse model of COVID-19 infection has been used here to discriminate between three clinical isolates, collected early on in the pandemic. One of these had a mutation which enhanced virulence in the transduced mouse: the D614G mutation in the SD2 region of the Gla1 isolate, which emerged in April 2020, is thought to have been selected for at the codon level to confer infectivity and transmissibility advantages [6]. In the transduced mouse model, we have reported here that Gla1 did indeed cause increased weight loss and increased bronchial inflammation, relative to the Vic1 isolate when a similar dose was administered. Gla1-infected mice also had a more pronounced staining for SARS-CoV-2 nucleoprotein in the lungs compared to Vic1 and Engl2 at 4 days post infection.

In the course of this study, we observed that after only a single passage in VeroC1008 cells, the Engl2 isolate underwent a 24 bp deletion in the furin cleavage site (FCS) [unpublished data], which has been reported by others [29]. This PRRAR mutation, covering the FCS, has previously been reported to result in reduced spike protein processing and reduced pathogenesis in a hamster model, resulting in the attenuation of SARS-CoV-2 [30]. In this study, the minimal weight loss induced by the Engl2 isolate, suggests an attenuation in virulence compared with the other isolates.

The transduced mouse model has proven to be very useful in discriminating between early viral variants of SARS-CoV-2, as demonstrated above. As time has elapsed, viral variants have arisen with the N501Y mutation in the S protein, which permits the virus to bind to murine ACE2 [31].

Here, we investigated early variants of SARS-CoV-2 that required the transduction of mice for susceptibility. Since spring 2020, further variants have emerged with significant mutations to provide WHO-annotated VOCs, with Omicron being the most recent VOC [1]. The Omicron variant (B.1.1.529) has 32 mutations in the S protein, 15 of which are in the RBD [29], which have been demonstrated to decrease the neutralisation capacity of convalescent sera raised to the S protein sequence in the parent SARS-CoV-2 virus [32,33,34]. The Omicron variant has recently been evaluated for virulence in K18-hACE2 transgenic mice, and also in C57Bl6, Balb/c and 129 mice, as well as older (>10 months) C57Bl6 mice, and unexpectedly induced little or no weight loss [35], unlike previous variants. Additionally, the viral titres of Omicron recovered from the nasal turbinates of infected mice were lower than for WA1/2020 [36]. The Omicron variant has been found to be attenuated in both mice and hamsters, compared with previous variants such as WA1/2020. This would seem to mirror observations in the human population where Omicron displays increased infectivity/transmissibility, but with reduced virulence, although whether this is due to the attenuation of the virus or increased immunity in the population, or both factors, is difficult to say [37]. The evidence suggests that Omicron is more likely to replicate in the upper rather than lower respiratory tract and there-fore is less able to cause lung disease, unlike the early isolates that we have studied here.

The ability of SARS-CoV-2 to evolve and mutate, whist also maintaining its infectivity and transmissibility, is of continuing concern. Indeed, current observations of the emergence of sub-lineages of Omicron (both BA.1, BA.2 BA.3, BA.4, BA.5) [38,39] are being closely monitored by the WHO [40]. Going forward, there will be a continued requirement for sensitive animal models, such as the transduced mouse, to screen early and rapidly for evidence of increasing virulence and potential vaccine escape. The transduced mouse model has the advantage, when wild-type mice are not naturally susceptible, of inducing consistent susceptibility rapidly and can be easily adapted for different viruses by just changing the receptor. The transduced mouse model was shown to discriminate between variants and has outputs such as lung titres and weight loss, that can be used in studies to determine the efficacy of medical countermeasures.

Content includes material subject to © Crown copyright (2022), Dstl. This material is licensed under the terms of the Open Government Licence except where otherwise stated. To view this licence, visit http://www.nationalarchives.gov.uk/doc/open-government-licence/version/3 (accessed on 29 June 2022) or write to the Information Policy Team, The National Archives, Kew, London TW9 4DU, or email: psi@nationalarchives.gov.uk.

## Figures and Tables

**Table 1:** Summary of isolates, doses and time-points collected from SARS-CoV-2 infected mice.

SARS-CoV-2 Variant	Study	Challenge Back-Titre Count (TCID_50_/mL)	Challenge Dose in 50 µL (TCID_50_)	Day Post-Infection of Sacrifice
Victoria-1 (Vic1)	1	5.5 × 10^3^	2.7 × 10^2^	4
Victoria-1	1	1.2 × 10^5^	6.1 × 10^3^	4
Victoria-1	1	7.9 × 10^5^	4.0 × 10^4^	4
Victoria-1	2	3.2 × 10^6^	1.6 × 10^5^	2, 3, 4, 7 and 10
England-2 (Engl2)	1	1.7 × 10^4^	8.7 × 10^2^	4
England-2	1	3.6 × 10^5^	1.8 × 10^4^	4
England-2	1	2.2 × 10^6^	1.1 × 10^5^	4
Glasgow-1(Gla1)	2	3.2 × 10^3^	1.6 × 10^2^	4
Glasgow-1	2	5.6 × 10^4^	2.8 × 10^3^	4
Glasgow-1	2	4.2 × 10^5^	2.1 × 10^4^	4

## Data Availability

All the data generated or analyzed during this study are included in this published article, and the additional files are available from the corresponding author upon reasonable request.
